# Evidence in the Japan Sea of microdolomite mineralization within gas hydrate microbiomes

**DOI:** 10.1038/s41598-020-58723-y

**Published:** 2020-02-05

**Authors:** Glen T. Snyder, Ryo Matsumoto, Yohey Suzuki, Mariko Kouduka, Yoshihiro Kakizaki, Naizhong Zhang, Hitoshi Tomaru, Yuji Sano, Naoto Takahata, Kentaro Tanaka, Stephen A. Bowden, Takumi Imajo

**Affiliations:** 1 Gas Hydrate Research Laboratory, Meiji University Global Front, 1-1 Kanda-Surugadai, Chiyoda-ku, Tokyo 101-8301 Japan; 2 Department of Earth and Planetary Science, University of Tokyo, 7-3-1 Hongo, Bunkyo-ku, Tokyo 113-0033 Japan; 3 Earth-Life Science Institute, Tokyo Institute of Technology, 2-12-1, Ookayama, Meguro, Tokyo 152-8550 Japan; 4 Department of Earth Sciences, Chiba University, 1-33 Yayoi-cho, Inage-ku, Chiba 263-8522 Japan; 5 Atmosphere and Ocean Research Institute, University of Tokyo, 5-1-5, Kashiwanoha, Kashiwa-shi, Chiba 277-8564 Japan; 6 Institute of Surface-Earth System Science, Tianjin University, 92 Weijin Road, Nankai District, Tianjin 300072 P.R. China; 7 School of Geosciences, University of Aberdeen, King’s College, Aberdeen, AB24 3UE Scotland; 8 Graduate School of Marine Science and Technology, Tokyo University of Marine Science and Technology, 4-5-7, Konan, Minatu-ku, Tokyo 108-8477 Japan

**Keywords:** Carbon cycle, Marine biology, Marine chemistry

## Abstract

Over the past 15 years, massive gas hydrate deposits have been studied extensively in Joetsu Basin, Japan Sea, where they are associated primarily with active gas chimney structures. Our research documents the discovery of spheroidal microdolomite aggregates found in association with other impurities inside of these massive gas hydrates. The microdolomites are often conjoined and show dark internal cores occasionally hosting saline fluid inclusions. *Bacteroidetes sp*. are concentrated on the inner rims of microdolomite grains, where they degrade complex petroleum-macromolecules present as an impurity within yellow methane hydrate. These oils show increasing biodegradation with depth which is consistent with the microbial activity of *Bacteroidetes*. Further investigation of these microdolomites and their contents can potentially yield insight into the dynamics and microbial ecology of other hydrate localities. If microdolomites are indeed found to be ubiquitous in both present and fossil hydrate settings, the materials preserved within may provide valuable insights into an unusual microhabitat which could have once fostered ancient life.

## Introduction

**Figure 1:**
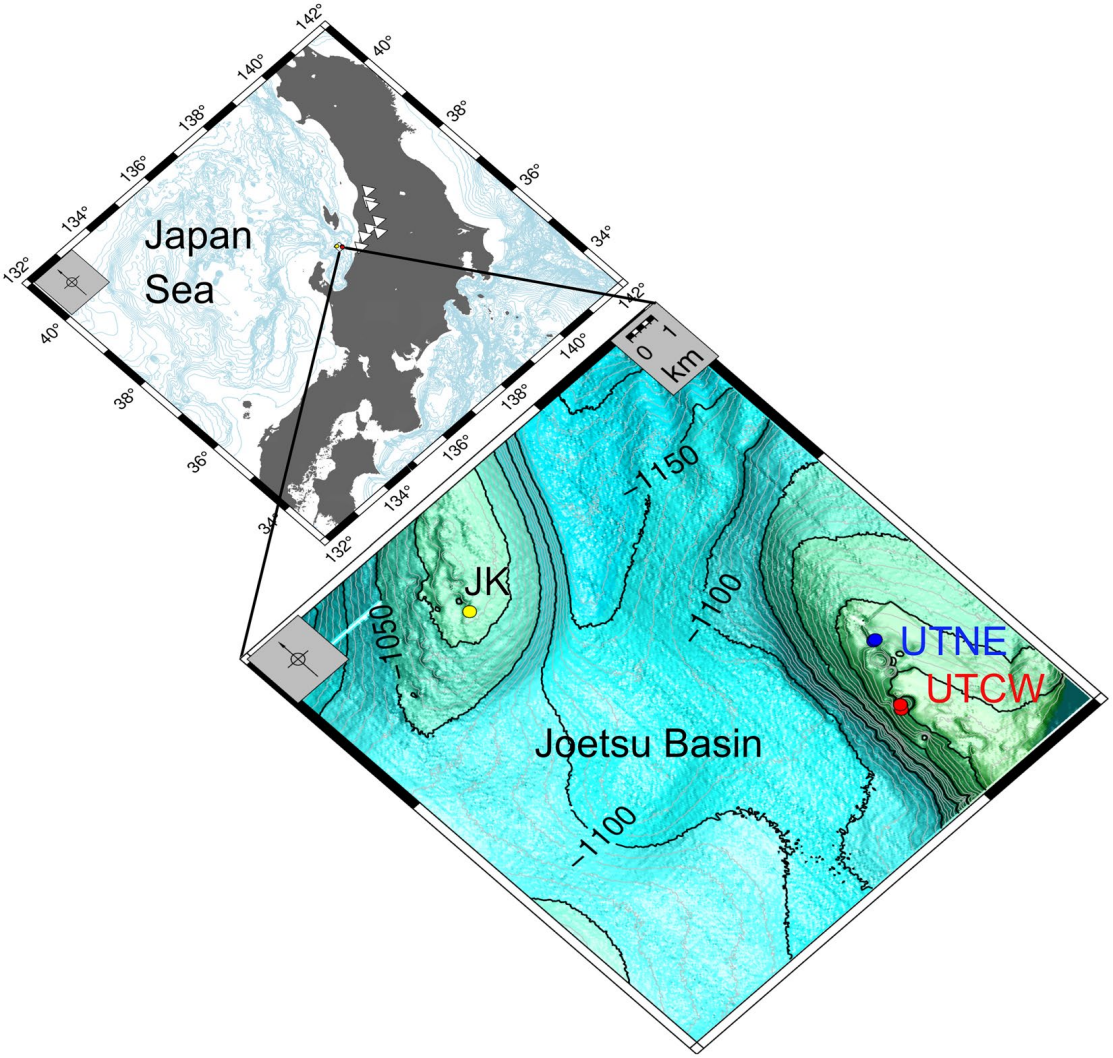
Microdolomite-bearing gas hydrate samples were collected by drilling at the crest to anticlinal structures in Joetsu Basin, located offshore Japan’s main island Honshu, between 900 m and 1000 m water depth. The sampling sites are in the general proximity of deep hydrocarbon wells located in Niigata Prefecture (white triangles). The Joetsu Knoll (JK) site is located on the southeastern crest of the knoll while the two Umitaka Spur sites were both located on the margins of giant pock marks in the northeast (UTNE) and central west (UTCW).

**Figure 2:**
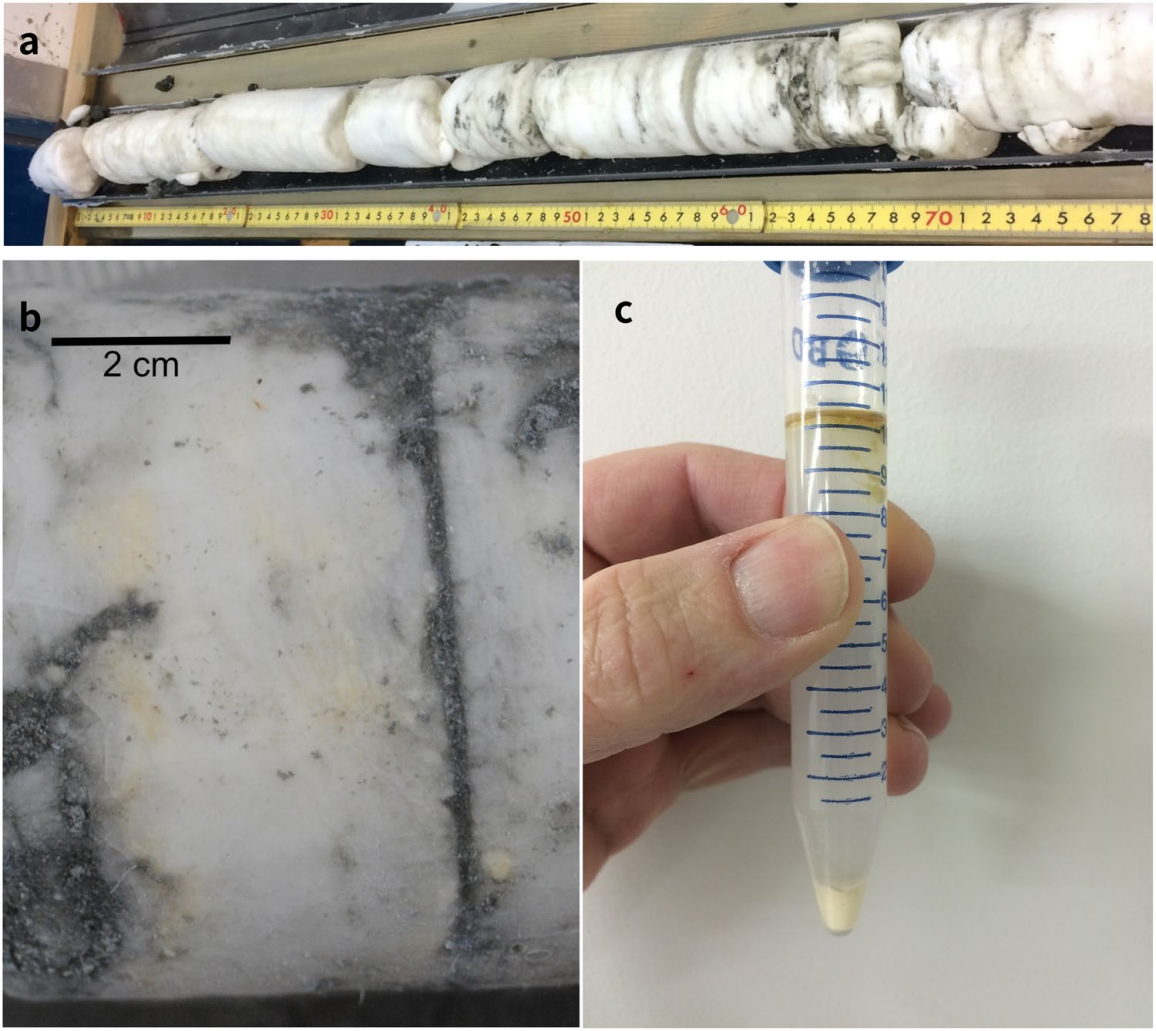
Hydrate before and after dissociation. (**a)** Core UTCW J25R 9 × 1–4 (68–70 mbsf) immediately after splitting the core liner on the ship. (**b)** Yellow patches within frozen hydrate. (UTCW J25R, 56 mbsf). The sediment-filled fracture is due to hydrate breakage during rotary drilling. The hydrate was broken and the pieces not containing sediment contamination were dissociated. (**c)** The same hydrate following dissociation and centrifugation, showing oil (top), a partially suspended mix of oil and water(middle) and a fine powder residue of loose dolomite grains (bottom).

The Umitaka Spur and Joetsu Knoll are well-known gas chimneys and sites of submarine methane seepage, situated on the western margin of the Japan Sea, offshore Niigata in Joetsu Basin **(**Fig. 1**)**^1,2^. The hydrocarbons from both sites are primarily thermogenic and accumulated as a result of the release of fluid overpressure that developed over the past 5 Ma in organic-rich Middle Miocene sediments^3,4^. In 2004, Umitaka Spur became the first site in the Japan Sea where gas hydrates were recovered and subsequent investigations have focused on: the chemistry of gas hydrates and seep gasses^5^, the formation of carbonate nodules and concretions (Methane Derived Authigenic Carbonates or MDACs)^6–9^ and the influence of gas seeps and gas hydrates on interstitial waters in the surrounding sediments^10–15^. Expeditions to the Joetsu Basin by the R/V Hakurei in 2014 (HR14) and R/V Poseidon in 2015 (PS15) recovered cores, several metres in length, that contained intact massive methane hydrate that was relatively uncontaminated by surrounding sediment **(**Fig. 2a,b**)**^16^. Ongoing research, including U/Th dating of associated MDACs^6,7^ and other sedimentological considerations^8^, suggest that these thick hydrate accumulations formed fairly rapidly at shallow sediment depths, and have been subsequently buried over time scales of tens of thousands of years.

Gas hydrates in the Joetsu Basin have been studied for over a decade, but it was not until routine hydrate dissociation experiments during the PS15 cruise that a cloudy residue was noted in the hydrate water **(**Fig. 2c**)**. This residue was found in hydrates dissociated shipboard and later in archived gas hydrate stored in liquid nitrogen from the same and previous cruises. Using powered X-ray diffraction, the residue was shown to comprise pure microdolomite, in the form of microscopic spheroidal aggregates recoverable without any special separation procedures **(**Supplementary Fig. S1, Supplementary Table S1**)**. Sampled hydrate sites **(**Fig. 1**)** include the margin of a large pock mark in the central western portion of Umitaka Spur (UTCW) and a hydrate-bearing site in the north-eastern portion of Umitaka Spur (UTNE). An archived sample from Joetsu Knoll (JK) cored during HR14 expedition was also included.

Here we show that microdolomites within the gas hydrates in the Joetsu Basin, Japan Sea, can grow by mineralising petrogenic carbon within isolated microhabitats provided by the methane hydrate. This microhabitat is unique in that the carbon is being taken from otherwise recalcitrant phases of carbon present within petroleum, and not via processes found outside the microhabitat such as sulphate reduction or anaerobic oxidation of methane.

## Results

### Growth, morphology and depth-related changes

**Figure 3:**
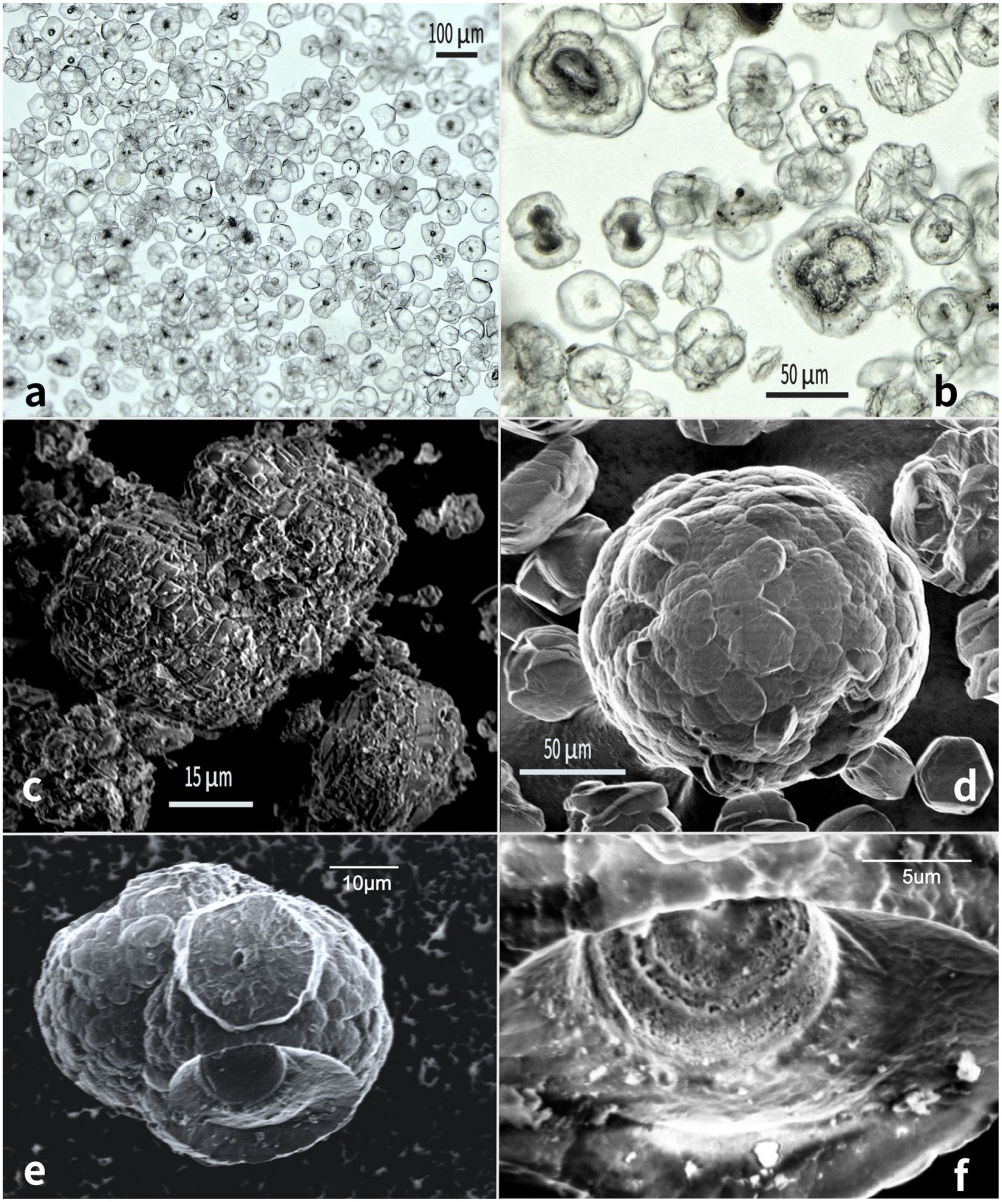
Mineral aggregates recovered from dissociated hydrate are relatively pure dolomite. (**a)** Light microscopy of single-grained dolomites showing dark inclusions (UTCW J25R, 53.9 mbsf, Mg/Ca = 0.91). (**b)** Single and paired “dumbbell” grains, showing layering in the internal dark portions (UTCW J22R, 28.7 mbsf, Mg:Ca = 0.92. (**c)** Shallow dumbbell grain (UTCW J21R, 12.2 mbsf, Mg:Ca = 0.74). Shallow grains (<20mbsf) show rough surfaces comprised of ~5 μm dolomite rhombs and low Mg/Ca ratios. (**d)** Deeper grains consist of smooth intergrown dolomite plates ~15 μm. The overall size of the deep grains ranges from 20 μm to > 150 μm and Mg:Ca ratios approaching 1 (UTCW J25R, 57 mbsf, Mg:Ca = 0.97). (**e)** Broken chain structure (UTCW J25R, 67.4 mbsf) shows smooth intergrowth of dolomite rhombs on the outer surface. (**f)** Close-up of previous grain showing concentric porous rings on the inside of the broken surface, possibly consisting of organic matter or residual fluid.

The Joetsu Basin dolomitic aggregates are easily distinguished from other minerals by their size, with diameters ranging from ~10 μm to ~150 μm (avg. diam. = 40 μm), and their distinctive morphologies e.g. “dumbbell pairs”, “chains”, or branching “cauliflower growths” (Fig. 3a–d**)**. These distinctive morphologies can be seen to be formed from conjoined microdolomite spheres. Dark-coloured material is present at the cores of the microdolomites, and broken chains reveal interconnected internal porous regions (Fig. 3e,f). Macroscopically, the microdolomite appears as a very fine white or light-yellow powder, as the conjoined growth-patterns rarely exceed several grains and are sufficiently dispersed within the hydrate matrix that spheroidal aggregates do not merge to form crusts, veins, or other larger cemented mineralisations (Fig. 2c**)**.

Although individual dolomite samples show some variation in the abundance of single grains, dumbbells, or cauliflower-shaped aggregates, the abundance of these morphologies does not change with depth. There is, however, an observable depth-related change in the outer texture and size of the aggregates. Hydrates sampled from <20 metres below seafloor (mbsf) contain aggregates of rough spheroidal angular dolomite rhombs **(**Fig. 3c**)**. The rhombs are ~5 μm across, and form spheroidal aggregates ~15–20 μm in diameter. There is a transition to smoother surface textures between 20 mbsf and 30 mbsf, such that dolomite rhombs sampled at >30 mbsf have intergrown dolomite surfaces **(**Fig. 3d**)**, comprising hexagonal plates or shield shapes ~10–15 μm across organized in spheroidal aggregates ranging from ~30–150 μm in diameter.

**Figure 4:**
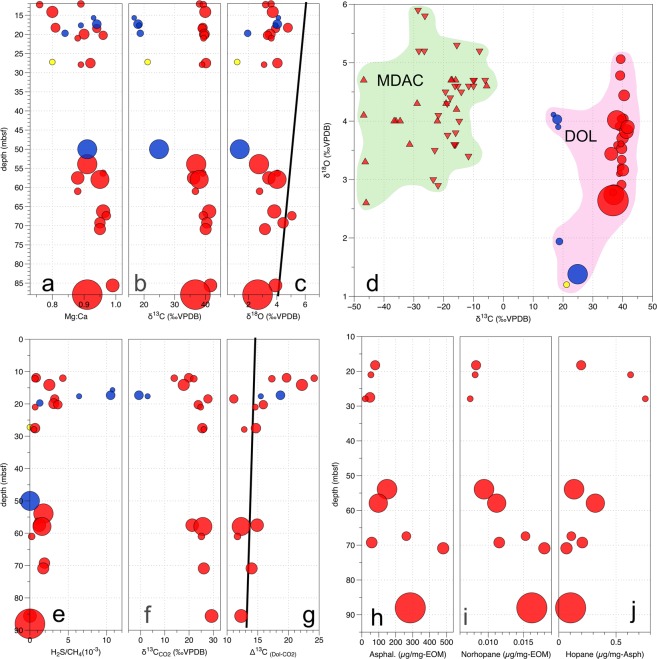
Depth profiles for recovered dolomite, hydrate gases, and oils found in hydrate. Point size is scaled to mean diameter of microdolomites (smallest at 19 µm and largest at 114 µm). Red = UTCW, blue = UTNE, yellow = JK. (**a–c)** The Mg:Ca ratios and stable isotopic composition of the dolomite aggregates. For δ^18^O, black line represents equilibrium values with seawater based on the geotherm. **(d)** Comparison of stable isotopic composition for Joetsu Basin microdolomites in this study (DOL) versus previously published MDACS from Umitaka Spur (upward triangles: Zhang *et al*.^9^; downward triangles:Hiruta *et al*.^8^). (**e–g)** Gas composition and stable isotopic composition for dissociated methane hydrate. The Δ^13^C values show the difference between dolomite and the hydrate CO_2_, illustrating the degree of disequilibrium that exists in shallow samples. Equilibrium values (solid line) based on the geotherm. (**h–j)** Organic chemistry of UTCW hydrate oil showing relative increases in refractory asphaltene and increases in C_29_ 25-norhopane at the expense of C_30_-hopane due to biodegradation.

Non-biogenic microdolomite aggregates with similar spheroidal and dumbbell morphologies have been produced in the laboratory at temperatures of >40 °C through direct precipitation from a gel of magnesium-rich amorphous calcium carbonate (MgACC) which quickly transforms to spheroidal proto-dolomite and eventually undergoes dewatering to produce microdolomite^17,18^. During this transformation, Mg:Ca ratios increase from 0.65 in pure MgACC to 1.00 in stoichiometric dolomite, where Mg:Ca is the molar ratio of the two corresponding elements in the dolomite. In order to see if the Joetsu Basin dolomites show any systematic change, Mg:Ca was determined through Rietveld refinement of the x-ray diffraction patterns^19^ and by applying the equation of Turpin *et al*.^20^. Grain diameter was determined through microscopy^21^ **(**Supplementary Tables S1, S2**)**. In general, both the grain size and average diameter of grains increase with depth **(**Fig. 4a). The smallest average grain diameter is 19 µm (UTNE at 16mbsf) while the largest average is 114 µm (UTCW at 88mbsf). At 22 µm, the Joetsu Knoll sample (JK at 30mbsf) is also quite small despite being much deeper than other small dolomite aggregates. The lowest Mg:Ca ratio is 0.76 (UTCW 12mbsf), while the highest is 0.99 (UTCW 86mbsf). There are, however some dolomites with Mg:Ca > 0.95 at depths as shallow as 20mbsf.

### Stable isotopic composition

Stable carbon isotope ratios can potentially indicate the carbon source of the microdolomites, particularly in the Joetsu Basin where the primary carbon pools have distinct isotopic compositions. The δ^13^C values for the microdolomites **(**Fig. 4b,d**)** are all positive, which is significantly different from the negative values associated with MDACS in the area^8,9^. Generally, dolomites which have positive δ^13^C values are the result of a carbon source related to methanogenesis which has subsequently undergone evaporation^22^. The Joetsu Basin hydrates have reported δ^13^C values ranging from −57.1‰ to −43.9‰VPDB in Joetsu Knoll and −36.6‰ to −34.6‰VPDB in Umitaka Spur^5^, both locations indicating a thermogenic source with some admixture of biogenic methane. Similarly, the δ^13^C values of methane reported for deep wells within the nearby Niigata gas fields range from −35‰ to −33‰VPDB^23,24^, overlapping with the range of values from Umitaka Spur. The reported δ^13^C values for dissolved inorganic carbon (DIC) in the interstitial waters of Joetsu Basin sediments range from +18.7‰ to +28.5‰VPDB at Joetsu Knoll and −4.9‰ to +41.4‰ at UTCW^15^; at both areas, the least positive values are shallow sediments near the sulphate methane transition (SMT), due to anaerobic oxidation of methane (AOM) whereas the most positive values are in the deeper sediments. Presumably the source of this DIC, which is enriched in ^13^C relative to ^12^C, is a deep source of residual organic that has undergone methanogenesis over long periods of time^3,4^. Porewaters with negative δ^13^C values for DIC are related to a combination of gas hydrate dissociation and AOM^8,24^.

During growth gas hydrate incorporates pore fluids resulting in residual porewaters with negative δ^18^O values^12,13,25^. The removal of porewater and the resulting dehydration is observed as salinity anomalies within interstitial water throughout surrounding hydrate locales of the Joetsu Basin^11,14,15^. Japan Sea bottom-waters in the Joetsu Basin are cold at 0.4 °C and the geothermal gradient at Umitaka Spur and Joetsu Knoll is 105 mK/m^26^. The temperature of the deepest samples at 90 mbsf, which is just above the gas hydrate stability zone (GHSZ) would be expected to be 9.9 °C. The δ^18^O value for dolomite in equilibrium with seawater down to this depth would range from +6.4‰ to +4.1‰VPDB^27^ (Supplementary Table S2). The dolomites show δ^18^O values to the left of equilibrium with seawater plotted as a solid line **(**Fig. 4c**)** due to isotopic fractionation between oxygen in the interstitial water and water-bound oxygen in the growing hydrate^13,28^. Both UTNE and JK dolomites show a greater degree of depletion of ^18^O than UTCW. If the extent of disequilibrium is taken as an indicator of the rate of growth, then most of the rapid growth of hydrates occurred at depths less than 20 mbsf. As hydrate is buried, the values again approach those of thermal equilibrium with seawater. Unlike the δ^13^C values, the δ^18^O values of MDACs in the UTCW area show considerable overlap with those values observed for the microdolomites **(**Fig. 4d**)**. The larger, deeper microdolomites show less positive values, particularly at UTNE, indicating that hydrate growth at depth continues and in doing so takes up water from the fluid inclusions in which the microdolomites also continue to grow.

### Non-hydrocarbon gases incorporated in hydrates

The Joetsu Basin hydrates contain both hydrogen sulphide and carbon dioxide^5,7^. Significant amounts of H_2_S (up to 10.8 mL/L-CH_4_) were found in hydrates between 10 mbsf and 20 mbsf. These depths coincide with the formation of small dolomite grains **(**Fig. 4e**)** and it may be that the presence of high sulphide concentrations in the hydrate contributed to the initial stages of microdolomite precipitation^29^. Carbon dioxide is also present, and the δ^13^C values for CO_2_ reach minimum values over the same interval **(**Fig. 4f**)** then gradually become more positive with depth as they reach equilibrium with the surrounding DIC-pool. The composition of non-hydrocarbon gases within the hydrate therefore seems to be influenced by AOM and sulphate reduction to some degree^5^, but only significantly so at depths of less than 20 mbsf. This is not the case for the microdolomites, as the δ^13^C of the UTNE microdolomites seems only to be influenced by AOM at shallow depths and not at all at UTCW **(**Fig. 4b**)**. Assuming the aforementioned temperature gradient, we calculated Δ^13^C_dolomite-CO2_ as a function of depth **(**Supplementary Table S2**)**^30^. Calculating the equilibrium values for the microdolomites indicates a high degree of isotopic disequilibrium with the CO_2_ for the hydrates from both UTNE and UTCW at <20 mbsf **(**Fig. 4g**)**. Even though AOM may influence the δ^13^C of CO_2_ in the hydrate, the primary source of carbon in the microdolomites must be ^13^C-enriched DIC, which can ultimately only derive from methanogenesis at greater depths or from some form of microbial activity which also produces CO_2_ within saline fluid inclusions in the gas hydrate.

### Oils, brines, and other impurities excluded during hydrate growth

It has been shown that rapid hydrate growth, at least in the case of synthetic hydrates, can lead to the temporary formation of encapsulated pockets of brine or finely dispersed saline fluid inclusions^31^. The Joetsu Basin hydrates recovered from the UTCW sites have yellow hydrate which, when dissociated, yields yellow oil which is emulsified in the clear hydrate water and microdolomite which settles to the bottom **(**Fig. 2c**)**. The recognition of distinct insoluble oil and water phases is important because when trapped in pockets and veins, water-in-oil emulsions both stabilize brines providing microbial habitats^32^ and could potentially serve as a spherical-template for the formation of mineral precipitates such as the spheroidal microdolomites. Evidence for microbial processes can be found in the chemical composition of the oils which exhibit alteration consistent with the subsurface degradation of petroleum^33^. Specifically, the higher carbon-number *n*-alkanes and steranes which are generally present in undegraded oil are notably absent, and instead biodegradation-products such as the 25-norhopanes have been formed from regular hopanes, and the oils relatively enriched in recalcitrant components such as asphaltene **(**Supplementary Fig. S3 and Supplementary Table S4**)**. The proportion of asphaltene, here taken as a more refractory organic component, becomes greater relative to total extractible organic matter (EOM) with depth, indicating that the biodegradation of the labile compounds is ongoing during burial especially in the upper 30mbsf **(**Fig. 4h**)** and is generally accompanied by an increase in the diameter of the microdolomite grains. Similarly, C_29_ 25-norhopane, which is formed directly from the biodegradation of C_30_ hopane increases with depth **(**Fig. 4i**)** and the amount of C_30_ hopane decreases relative to asphaltene.

### Internal chemistry of dolomite grains

**Figure 5:**
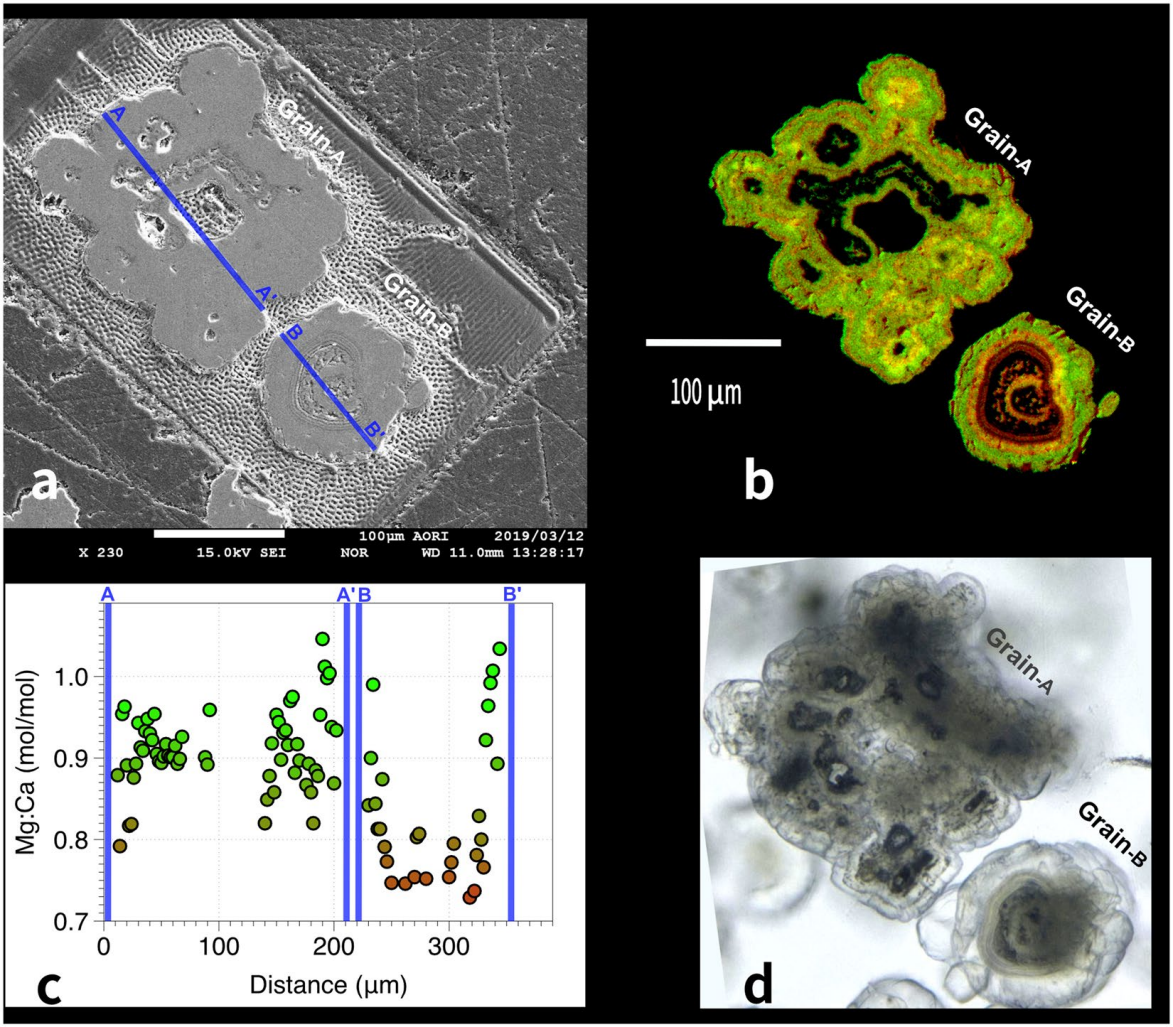
**(a)** SEM scan of a composite “cauliflower” (Grain-A) and a single grain dolomite aggregate (Grain-B) from UTCW Site J25R (57.9mbsf) following elemental mapping. Blue line shows where quantitative elemental mapping was carried out at 2 μm intervals using EPMA. (**b)** EPMA mapping shows that areas high in Mg (green) are concentrated on the transparent outer surface, while areas to the center of the grains are relatively more enriched in Ca (red). (**c)** Quantitative scanning of Mg:Ca ratios across shows much lower Mg:Ca ratios near the dark cores and internal voids, particularly in the single-grain sample. (**d)** Transmission light microscopy shows concentric layering around dark core material.

The majority of the microdolomites show zonation as do other microbial dolomites^22^; the Joetsu Basin microdolomites have outer rims with high Mg:Ca ratios and are optically clear while the darker central cores have lower Mg:Ca ratios, sometimes approaching Mg:Ca = 0.7 **(**Fig. 5**)**. This may reflect that hydrate growth is initially rapid in shallow sediments and leads to less-ordered dolomites. However, during hydrate burial the dolomites grow slowly until the overall Mg:Ca ratio approaches 1 **(**Fig. 4a**)**. Prior to analysis, and during the sample preparation, it was also noticed that some of the grains still contain fluid inside **(**Supplementary Fig. S2**)** which, if left to dry, formed secondary minerals on the surfaces of the polished microdolomites. EPMA showed that these grains have high Na and Cl in the cores, presumably trapped saline water.

### Internal microbial content of dolomite grains

**Figure 6:**
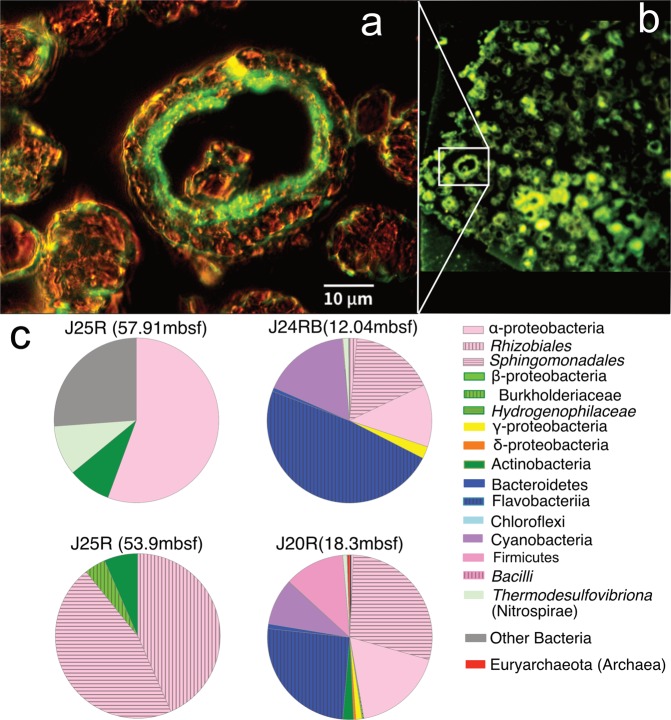
(**a)** Epifluorescence imagery of dolomite grain showing internal presence of microbial DNA. In this case, the dolomite crystals forming the aggregate appear amber-colored while nucleic acid in the microbial material fluoresces due to SYBR-green dye (UTCW J24RB, 12.0 mbsf). (**b)** Fluorescence of the same sample (white box) showing high density of microbial DNA contained within surrounding microdolomite grains. (**c)** Pie charts showing the relative abundance of microbial phyla within deep and shallow samples.

Epifluorescence microscopy of DNA-stained microdolomites indicated high concentrations of microbial DNA in two samples from shallow depths (less than 30 mbsf), and lesser concentrations in two samples beneath 30 mbsf. Despite differences in DNA-concentrations, both shallow and deep microdolomites could yield sufficient extractible DNA for 16S rRNA phylogenetic analysis (Supplementary Table S5). Epifluorescence microscopy showed that DNA lined the inner surfaces and cores of the spheres (Fig. 6), strongly indicating that phylogenetic information for the microdolomites pertains to their internal microhabitat.

It is notable that the microdolomites mostly lack sulphate reducing bacteria and ANME archaea that are associated with gas hydrate mounds and shallow sites of methane seepage^34–36^. A single sample (J20R 18.5 mbsf) had 0.5% archaea and 0.5% δ-proteobacteria (possible sulphate reducing bacteria), but aside from this the interior of the microdolomites appears to be a microhabitat distinct from that typically found at shallow sites of methane seepage. Instead, phylogenetic analysis suggests the microdolomites grew in a microhabitat similar to that hosted by deep gas hydrates^37^ and by marine oil spills^38^. For example, *Sphingomonadales* is present in all samples, and there is a notable predominance of α-proteobacteria in the deepest microdolomites (*Rhizobiales* makes up 50% of the microbial abundance in J25R 53.91 mbsf, compared to 17.9% for other α-proteobacteria in shallower samples). Both *Sphingomonadales* and *Rhizobiales* are reported oil-degraders.

However, there are differences to the communities reported from marine oil spills: β-proteobacteria, including *Burkholderiales*, which commonly occur in oxic-seawater near oil seep sites^38^ are absent, while γ-proteobacteria are of low abundance in shallow samples and absent in deeper samples. The low abundance of γ-proteobacteria and greater relative abundance of α-proteobacteria in the deepest samples is consistent with α-proteobacteria supplanting γ-proteobacteria during the later stages of petroleum degradation, after the lighter substrates have been removed^39^.

From the perspective of the formation of microdolomites, perhaps the most striking difference between the deep and shallow microbial communities is the abundance of *Bacteroidetes;* in particular *Flavobacteriia* which is present in the shallower samples (48.3% at 12.04 mbsf and 25.3% at 18.25 mbsf) and completely absent from the deeper samples. Because *Flavobacteriia* breaks down complex macromolecules including oils^39^, and produces extracellular polymeric substances that initiate the formation of spherulitic microdolomite^40^, *Flavobacteriia* likely plays a key role initiating the formation of microdolomites at shallow depths. Some strains of *Flavobacteriia* have a light-yellow colour^39^ and this may account for the yellow colour of some microdolomites and oils. Because both hydrate and sediment in Joetsu Basin samples contain oil, the sediments also contain some similar organotrophs^41^, as do sediments above deep gas hydrate on the Pacific side of Japan^42^, yet the formation of authigenic carbonates in the sediments appears to be predominantly a consequence of shallow ANME and SRB.

Of final note is the Phylum *Firmicutes*, including Class *Bacilli*, which is present in the shallow microdolomites, but more generally associated with hypersaline anoxic environments^37^. The Phylum *Firmicutes* is present at 11.8% in one shallow sample (J20R 18.25 mbsf), but the Class Bacilli is found only in trace amounts in both shallow samples and not at all in the deeper samples. In one of the deeper microdolomite samples (J25R 57.91 mbsf), the Phylum Firmicutes, Class *Thermodesufovibrionia* makes up 9.8% of the microbial distribution and its presence is most likely associated with the degradation of long-chain alkanes and fatty acids^43^.

## Discussion

Authigenic carbonates are often associated with gas hydrates and gas chimneys, but the microdolomites in the residue from dissociated Joetsu Basin hydrate differ from MDACs in several respects. The first major difference is mineralogy. The MDACs found at the Umitaka Spur and Joetsu Knoll gas chimneys all comprise aragonite or high-Mg calcites^8,9^, and occur as concretions or nodules of cemented sediment. To date, microscopic observation of the MDACs in Joetsu Basin has revealed the development of micritic carbonate and carbonitic microspar on sediment surfaces, but until this study had not found spherulitic aggregates of microdolomite. Hydrate Ridge located offshore Oregon is similar the Joetsu Basin Sites in a number of regards, including the presence of yellow oil-containing hydrate^44^, and is known to host carbonate “clathrites” that developed in very close proximity to massive hydrate and which also consist of high-Mg calcitic and aragonitic sediment cementation^25^ similar to the Joetsu Basin MDACs^8,9^. In contrast, the carbonate present inside of the Joetsu Basin hydrates is exclusively microdolomite. The second difference is in the microbial communities associated with the carbonate growth which, in shallow marine sediments, is related to the availability of porewater sulphate. Such settings, which are external to the hydrate surfaces, give rise to a consortium of ANME Archea and SRB as has been documented in Hydrate Ridge^35^. Given the absence of available sulphate inside of fluid inclusions within the hydrate, the microbial content preserved in the microdolomites is lacking in ANME and SRB. Although methane is available in abundance within the hydrate, the microbial communities inside of the Joetsu Basin microdolomites are primarily organotrophs which rely on complex macromolecules as a metabolic source. Other key differences between conventional MDACs and the microdolomites, such as stable isotopic composition and growth habit, are intimately tied to the first two differences. The effect of anaerobic methane oxidation, for example, does not seem to influence the δ^13^C of the microdolomites, even though it has produced very negative δ^13^C values in the nearby MDACs **(**Fig. 4d**)**. The spheroidal growth habit observed with the microdolomites which appears to be the consequence of extracellular polymeric substances produced by *Flavobacteriia*, is also not observed in nearby MDACs.

At first glance, the hydrate microdolomite grains seem perhaps similar to oolitic carbonates found in fossil hydrocarbon seep settings in Japan^45^ and elsewhere^46^. As with Joetsu Basin MDACs, however, these oolitic growths are related to the production of dissolved inorganic carbon (DIC) through microbial activity carried out by ANME and SRB and have negative δ^13^C values. Also, the microdolomites differ significantly in size, not exceeding 200 μm in diameter, whereas the oolitic growths which developed in the aforementioned seep settings are about an order of magnitude larger in diameter and often consist of acicular aragonite which coats pre-existing grains.

By considering the microdolomites as a microhabitat or micro-environment, better analogues can be found based on chemical characteristics, rather than facile comparisons to geological settings. For example, highly evaporative settings may develop saline anoxic lagoons or sabkha environments where carbonate authigenesis is locally mediated by the extracellular polymeric substances produced though microbial activity^22,40^. Spheroidal microdolomite aggregates of similar size and growth habit have been shown to develop in Lagoa Vermelha and Brejo do Espinho, Brazil^47,48^. Petroleum seepage sites in Kuwait (Eocene and Quaternary sediments) have been shown to host hydrocarbon-related microdolomites^49^. Microbially-derived spheroidal microdolomites have also been reported, along with evidence of previous gas hydrate occurrences, in the Tertiary-age seep sites of Monferrato, Italy^50^. In these settings, the formation of dolomite is favoured at high salinities, petroleum provides the organic substrates, and microbial processes which ensue both oxidise large organic molecules and mediate the formation of dolomite. In the case of Joetsu Basin, a hypersaline micro-environment would be generated by the rapid growth of the surrounding hydrate^11,12,15^ coupled with the drawdown of water and the formation of saline water inclusions, and with hydrocarbons seeping up from the underlying petroleum system^3,4^.

Our results suggest the development of a microbiome inside of the saline fluid inclusions that form during rapid growth of massive hydrates within gas chimneys. This rapid hydrate growth has led to the exclusion of oily, saline pockets inside of the hydrate. During the exclusion, a water-in-oil emulsion may form which limits the migration of saline waters out of the hydrate and provides a suitable medium for the biodegradation of oils to occur. The uptake of water by the growing hydrate further concentrates the residual brines, which reach saturation with respect to dolomite, while increasing the concentration of dissolved nutrients available to microorganisms.

Organotrophic microbes, in particular *Flavobacteriia*, metabolize the oils, producing extracellular polymeric substances that are suitable for the formation of spherulitic microdolomite^40,51^. In addition, the same organotrophic microbes produce DIC which is enriched in ^13^C, similar to the DIC which has migrated upward from depth, along with the oils and methane. Dolomite precipitation initiates around extracellular polymeric substances excreted by the microbes and, since the microbial activity was centred around the oil-covered water droplets, saline water becomes trapped inside microdolomites. Although microbial activity appears to be focused at depths less than 30mbsf, the microdolomites continue to grow as they are buried with the hydrate, increasing in diameter over time and developing dolomite coatings with higher Mg:Ca ratios.

Although the presence of microdolomites has only recently been observed in Joetsu Basin, future research will focus on whether or not they are present in other environments, such as shallow permafrost or deep pore-filling hydrates. Given that the conditions leading to shallow hydrate growth are not unique to the Japan Sea, it is also likely that this unusual microbiome is present in other settings, including permafrost hydrate, other offshore hydrate settings, and areas that preserve sedimentological evidence of fossil gas seeps. Given that spheroidal microdolomites can potentially encapsulate seawater and organic matter inside, such grains may potentially preserve valuable information regarding ancient life.

## Methods

### Sample collection

The contents of the clear polycarbonate liners were inspected as soon as the core arrived on deck. Where massive hydrate was observed, 10–20 cm whole-round sections were cut immediately and preserved in liquid nitrogen for on-shore research. Remaining core was cut into 1 m sections for core description, including intervals which also included nearly pure hydrate.

Identical procedures were used to disassociate gas hydrate on-shore and shipboard; the outer hydrate portions removed, 20–30 cc of clean hydrate dissociated in 50 cc syringe (preformed in a fume hood due to hydrogen sulphide), and evolved gases collected in aluminium polymer bags with Teflon stopcocks. The remaining liquid phase was agitated with a vortex stirrer, transferred to a 50 cc tube and centrifuged. A Pasteur pipette was used to collect oil residues from the surface, the liquid phase was decanted and a solid phase at the bottom recovered, rinsed and cleaned. (Residue was centrifuged twice with 18 MΩ deionized water, final rinse with ethanol prior to drying at 40 °C).

### X-Ray Diffractometry (XRD)

The hydrate-residue had a grain-size  that was sufficiently fine that it did not need to be ground for powder XRD. XRD was carried out on a Rigaku Ultima IV with the following settings: rotation: 40 RPM, 2θ-range: 5° - 85°, 2θ-step size: 0.02°, scan step speed: 0.8 s, Generator: 40 kV and 30 mA. The stoichiometric ratio of Mg:Ca in the microdolomites was determined from the XRD patterns using the method of Turpin^20^ (Table S1). Mineralogical abundances were determined through Reitveld Refinement (Table S2) using Profex software^19^. Of the 39 samples, 28 had >15% dolomite and were taken for further analysis (11 had a high marine clay-content and were not analysed further).

### Microscopy

External features of uncoated microdolomites were observed with a Keyence VE-8800 Real Surface-View Scanning Electron Microscope (3 kV to 8 kV). Internal details of the microdolomites were observed using a Keyence Digital Microscopy System and polished resin blocks. Resin blocks were prepared by embedding the microdolomite in Luff Araldite resin (Polysciences), placing the resin under low-vacuum to remove air-bubbles and, once hardened, polishing with plastic lapping film (3M). This procedure was carried out with dry lapping film to avoid dissolution of grain materials. To image the internal presence of microbial DNA, the microdolomite samples were sectioned with a diamond-blade microtome and stained with SYBR green. Confocal epifluorescence microscopy was done with an Olympus BX51 fluorescence microscope equipped with an Olympus DP71 charge-coupled device (CCD) camera.

#### Elemental mapping and electron probe microanalysis

Elemental mapping was carried out on Pd/Pt-coated polished samples using JEOL Superprobe (XA-8230) with the following instrument parameters; Spot mode, AccV = 15 kV, ProbC = 50 nA, dwell time = 60 msec/pixel. Samples were polished as described above the microscopy of internal details. Elemental mapping provided intensity values that were mapped using ImageJ software^52^. For quantitative determination of the ratio Mg:Ca, analyses were taken at a 2 μm frequency along linear transects that crossed grain surfaces. The following calibration standards were scanned before and after each transect to correct for instrument drift and permit the calculation of relative intensities: CaSiO_3_ for calcium and MgO for magnesium.

#### Stable isotopic composition of microdolomites

The carbon and oxygen stable isotopic the microdolomites was determined using a ThermoFisher GC-MS (MAT 243) equipped with a GasBench system. A small amount of powdered microdolomite (200–300 μg) was digested overnight in 100% H_3_PO_4_ at 80 °C. The resultant CO_2_ gas was then injected into the IRMS automatically for carbon and isotope analysis. The δ^13^C and δ^18^O values were calibrated using NBS19. A dolomite acid fractionation factor for δ^18^O was calculated according to Rosenbaum and Sheppard^53^.

### Hydrate gas composition

The gas from hydrate dissociation was collected in polymer-coated aluminium gas-collection bags (GL Sciences) Gas standards were prepared from 99.9% CH_4_ (GL Science) and the methane concentration was determined by injecting 0.5 mL of hydrate gas into a GC-FID (Shimadzu GC14B) with an alumina column (60/80, GL Science). A second sample split of 0.5 mL was injected into a GC-FPD (Shimadzu GC-2014S) equipped with a β, β-ODPN 25% Uniport HP 6/80 column in order to determine H_2_S concentration. Calibration standards were prepared from a 10% H_2_S gas (Takachiho Chemical Industrial). The results of the two analyses were used to calculate a molar ratio of H_2_S/CH_4_. Stable isotopic analysis of the δ^13^C of CO_2_ in the dissociated hydrate was carried out by first transferring gas into a vacuum chamber with a Pfeiffer Prisma 200 quadrupole mass spectrometer to analyse gas composition. Samples with less than 10% air contamination (based on O_2_ content), were then passed through a liquid-nitrogen cold trap in order to separate CO_2_. Carbon isotopes were then measured for the CO_2_ using an IsoPrime 100 mass spectrometer.

### Oil analysis

Biomarker analysis was carried out on a 6890N Network GC system interfaced to a 5975 inert mass selective detector with a PTV injector. Oil was extracted from aliquots oily hydrate water by liquid-liquid extraction. Extractions were performed by shaking water in the presence of dichloromethane and the products of each stage were combined. A quantitation standard of 5β-cholane was added. Extracts were analysed by GC-MS using the following method: PTV injector (300 °C) operating in splitless mode; GC temperature program was as follows; 60 °C to 120 °C at 20 °C/min then from 120 °C to 290 °C at 4 °C/min. The column was a Greyhound GC-5 (HP-5 equivalent phase; 30 m length, 250 µm ID and 0.25 µm film thickness). The MS was operated in sim mode (less than 20 ions & dwell time less than 40 ms). Compounds were identified by reference to well-characterized samples of biodegraded oil from the Niger Delta^54^. Surface enhanced Raman spectroscopy of asphaltene was done using a BWTek i-Raman Pro fitted with a 532 nm light source and mounted on 20 × video-microscope and employing a gold-coated glass substrate. A small aliquot of extract and asphaltene-quantitation standards dissolved in dichloromethane were absorbed on the gold surface. Surface enhanced Raman spectra were collected by accumulating 1000 spectra over a 300 ms duration in the range 200–2000 cm^−1^, with the 1200 to 1800 cm^−1^ region used. Laser spot size was approximately 2–4 μm and laser power was 40–60% (<13 mW delivered to the sample). Quantification was performed using the procedure in Bowden and Taylor^55^; deconvolution with entire spectra of asphaltene and interfering compounds and quantification of asphaltene.

### Microbial content of microdolomites

As previously described^56^, 0.1 g of crushed microdolomite sample was incubated at 65 °C for 30 min in 150 μL of alkaline solution (pH 13.5, 75 μL of 0.5 N NaOH, and 75 μL of TE buffer including 10 mM Tris-HCl and 1 mM EDTA). Following centrifugation at 5,000 g for 30 s at room temperature, the supernatant was neutralized with 750 μL of TE buffer and 150 μL of 1 M Tris-HCl (pH 6.5). The DNA was concentrated by ethanol precipitation from the DNA-bearing solutions (pH 7.0–7.5) and the DNA precipitate was dissolved in 50 μL of TE buffer (pH 8.0). Sequencing and phylogenic analysis was carried out with an Illumina MiSeq sequencer. Using the primers Uni530F and Uni907R^57^, including TruSeq adapter sequences and 7-mer index^58^ 16S rRNA gene sequences was amplified by PCR using LA taq polymerase (Takara-Bio, Inc., Japan) for Illumina MiSeq sequencing. Thermal cycling was performed with 35 cycles of denaturation at 96 °C for 20 s, annealing at 58 °C for 45 s, and extension at 72 °C for 120 s. The PCR products were subjected to electrophoresis on 1.5% agarose gels and purified using the MinElute Gel Extraction Kit (Qiagen). The purified PCR products were mixed and used as templates for sequencing by MiSeq Genome Analyzer using MiSeq Reagent Nano Kit v2 with 500 cycles following the manufacturer’s instructions (Illumina, USA). Raw reads were processed using QIIME2^59^ for Phylotype composition analyses, including quality assessment, quality trimming, chimera detection and OTU clustering (97% cut-off). Initial taxonomic assignment was determined using a BLASTn-based similarity search against a nucleotide collection consisting of sequences from GenBank, European Molecular Biology Laboratory (EMBL), DNA Data Bank of Japan (DDBJ), and Reference Sequence (RefSeq)^60^.

## Supplementary information

Supplementary Dataset 1.
