# Supplementary material for: Evidence in the Japan Sea of microdolomite mineralization within gas hydrate microbiomes
Source: Sci Rep. 2020 Feb 5;10:1876. doi: 10.1038/s41598-020-58723-y (PMC7002378; doi:10.1038/s41598-020-58723-y)
Supplement: Supplementary file 1 — Supplementary Dataset 1. [file 41598_2020_58723_MOESM1_ESM.docx]

**Supplementary Figures and Tables for:**

Evidence in the Japan Sea of microdolomite mineralization within gas hydrate microbiomes

**Glen T. Snyder1*, Ryo Matsumoto1, Yohey Suzuki 2, Mariko Kouduka^2^, Yoshihiro Kakizaki1,2, Naizhong Zhang^1,3^, Hitoshi Tomaru4, Yuji Sano5,6, Naoto Takahata5, Kentaro Tanaka5, Stephen Bowden^7^ and Takumi Imajo8**

*1Gas Hydrate Research Laboratory, Meiji University Global Front, 1-1 Kanda-Surugadai, Chiyoda-ku, Tokyo 101-8301, Japan*

*2Department of Earth and Planetary Science, University of Tokyo, 7-3-1 Hongo, Bunkyo-ku, Tokyo 113-0033, Japan*

*3Earth-Life Science Institute, Tokyo Institute of Technology, 2-12-1, Ookayama, Meguro, Tokyo 152-8550, Japan*

*4Department of Earth Sciences, Chiba University, 1-33 Yayoi-cho, Inage-ku, Chiba, 263-8522, Japan*

*5Atmosphere and Ocean Research Institute, University of Tokyo, 5-1-5, Kashiwanoha, Kashiwa-shi, Chiba, 277-8564, Japan*

*6Institute of Surface-Earth System Science, Tianjin University, 92 Weijin Road, Nankai District, Tianjin, 300072, P.R. China.*

*7School of Geosciences, University of Aberdeen, King’s College, Aberdeen, AB24 3FX, Scotland.*

*8Graduate School of Marine Science and Technology, Tokyo University of Marine Science and Technology, 4-5-7, Konan, Minatu-ku, Tokyo 108-8477, Japan*

**Corresponding author*

**Figure S1.** Stacked diffractogram showing similar composition in all of the dolomite samples.


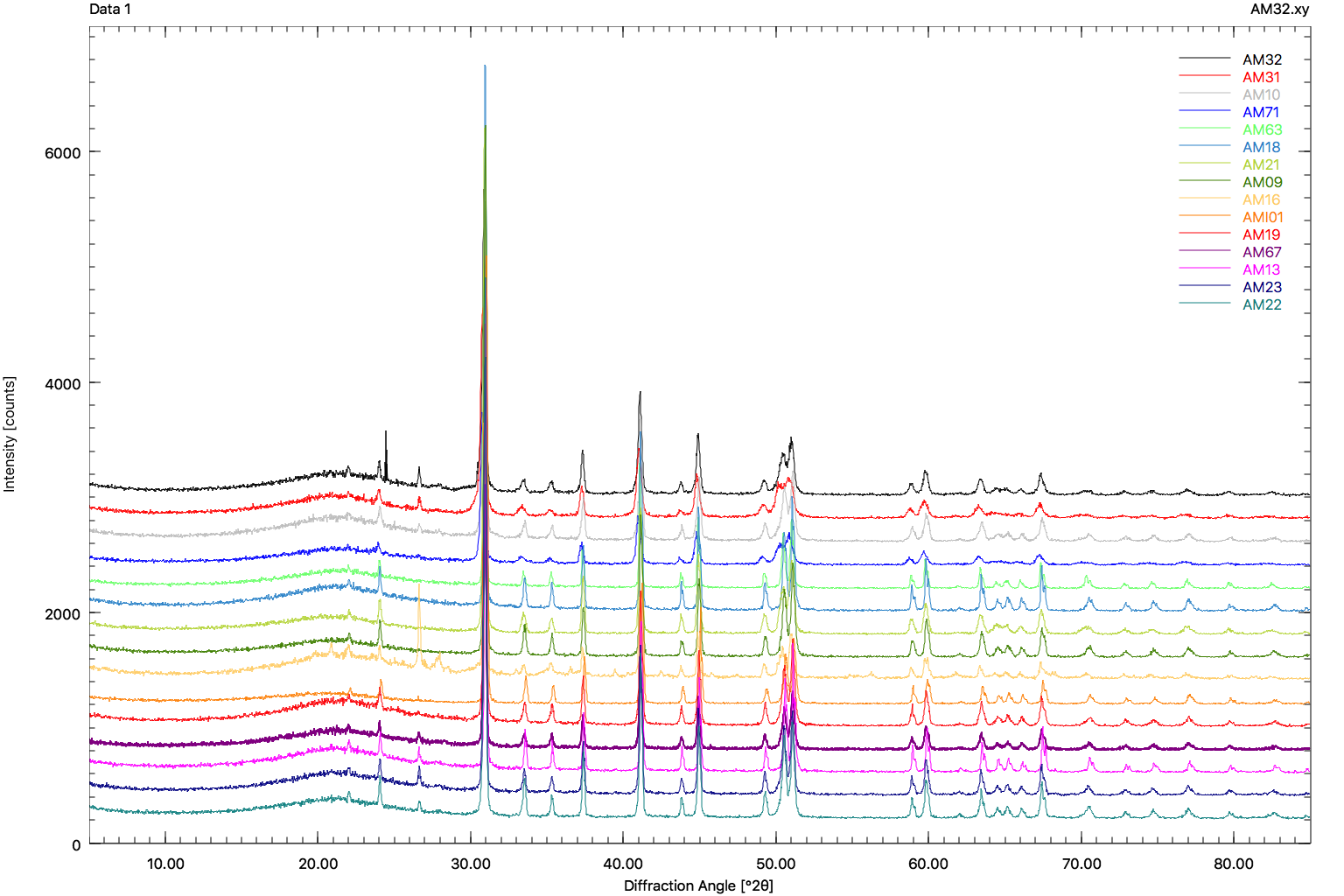


**Figure S2.** Evidence for the presence of saline fluids within the dolomite grains from UTCW Site J25R. **a)** Water droplets formed on the on the surface of the embedding resin during sample polishing with dry plastic abrasive film (67.4 mbsf). **b)** Light microscopy of embedded grains, 2 weeks after polishing, showing the development of crystals on the sample surface (J25R 56.27mbsf). **c)** SEM image of sample prepared for EPMA showing newly-formed crystals on the sample surface (57.51mbsf). **d)** SEM image of polished surface for EPMA (67.40 mbsf). **e)** Composite elemental mapping showing dolomitic outer grain surfaces with highest Mg (green) content on the outer grain surfaces, slightly greater Ca (blue) content in the inner mineral surface and high Cl concentrations (blue) in the core. Na (not shown) was also high in the grain cores (67.40 mbsf). **f)** Individual elemental maps used in the composite.


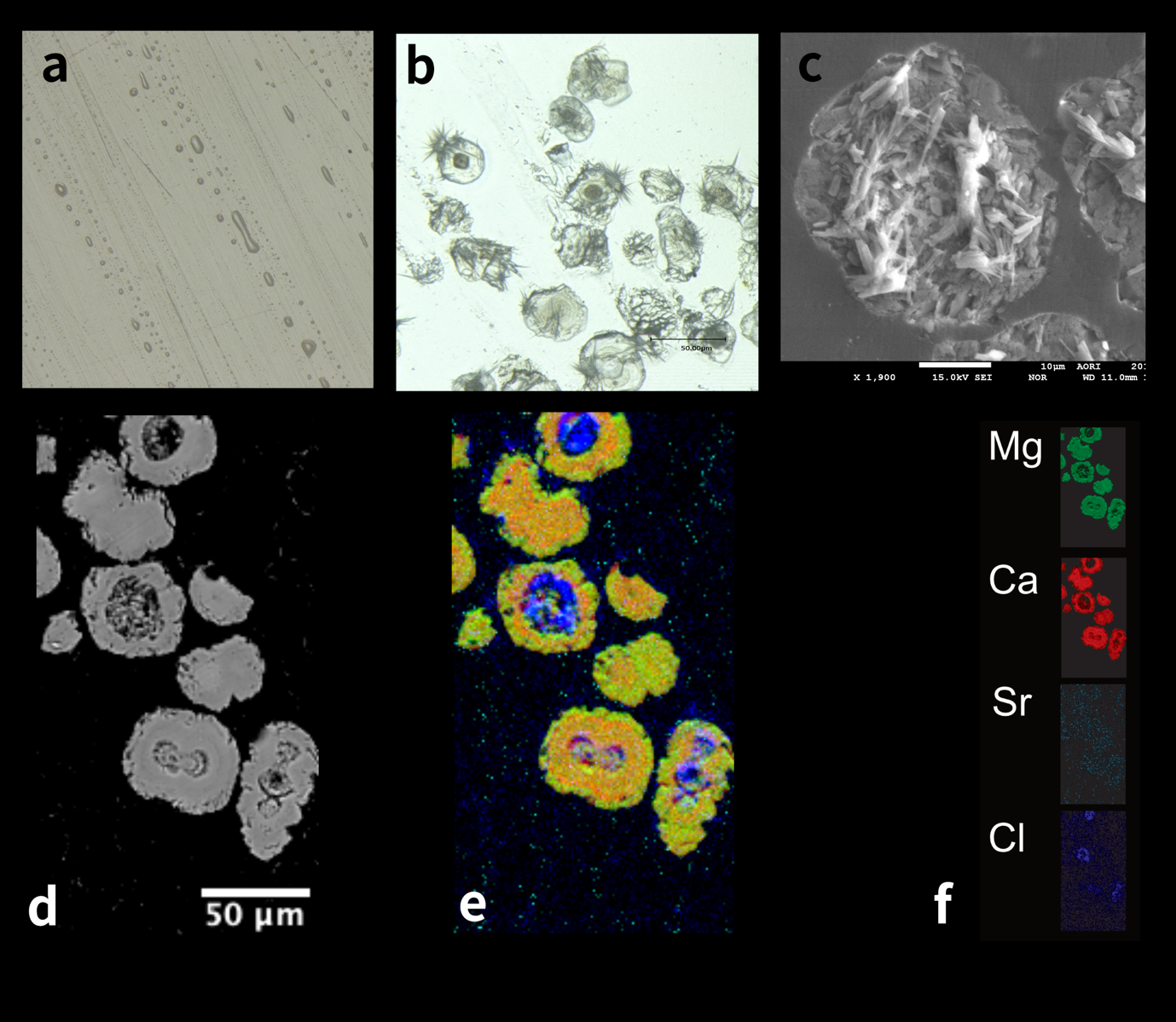


**Figure S3**. a) 217 and 259 *m/z* Ion chromatogram showing the presence of Diasteranes and absence of steranes in oil from UTCW J25R (67.4mbsf). b) 191 and 177 *m/z* Ion chromatogram for the same oil showing the presence of hopanes and 25-norhopanes.

**Table S1: Locations, Reitveld Refinement results, and average. grain diameter.**

| Sample ID | Location | Hole | mbsf | Mg/Ca | Dol | Cal | Qtz | Kao | Ill | Chl | Alb | Diam. |
| --- | --- | --- | --- | --- | --- | --- | --- | --- | --- | --- | --- | --- |
|  |  |  |  |  |  |  |  |  |  |  |  | (um) |
| ***Joetsu Knoll (JK)*** | | | | | | | | | | | | |
| AM42 | JK | J04RB | 27.22 | 0.80 | 0.53 | 0.00 | 0.11 | 0.14 | 0.07 | 0.07 | 0.10 | 22.4 |
| ***Umitaka Spur Central West (UTCW)*** | | | | | | | | | | | | |
| AM28 | UTCW | J21R | 11.92 | 0.80 | 0.57 | 0.02 | 0.07 | 0.11 | 0.06 | 0.04 | 0.14 | 34.4 |
| AM32 | UTCW | J24RB | 12.04 | 0.89 | 0.83 | 0.01 | 0.02 | 0.07 | 0.01 | 0.02 | 0.03 | 26.3 |
| AM88 | UTCW | J21R | 12.22 | 0.76 | 0.50 | 0.01 | 0.09 | 0.14 | 0.05 | 0.07 | 0.15 | 26.1 |
| AM31 | UTCW | J24RB | 14.12 | 0.80 | 0.82 | 0.01 | 0.01 | 0.07 | 0.03 | 0.04 | 0.02 | 43.7 |
| AM71 | UTCW | J20R | 18.25 | 0.81 | 0.84 | 0.00 | 0.00 | 0.08 | 0.02 | 0.04 | 0.01 | 35.3 |
| AM10 | UTCW | J20R | 18.45 | 0.94 | 0.83 | 0.01 | 0.01 | 0.07 | 0.03 | 0.02 | 0.03 | 32.9 |
| AM11 | UTCW | J20R | 19.93 | 0.90 | 0.78 | 0.01 | 0.00 | 0.13 | 0.03 | 0.04 | 0.02 | 39.1 |
| AM68 | UTCW | J20R | 20.24 | 0.96 | 0.77 | 0.01 | 0.00 | 0.13 | 0.03 | 0.04 | 0.03 | 33.2 |
| AM80 | UTCW | J22RC | 21.00 | 0.88 | 0.35 | 0.00 | 0.13 | 0.13 | 0.06 | 0.11 | 0.21 | 23.3 |
| AM82 | UTCW | J22RC | 27.50 | 0.92 | 0.78 | 0.01 | 0.01 | 0.09 | 0.01 | 0.06 | 0.05 | 37.4 |
| AM81 | UTCW | J22RC | 27.90 | 0.89 | 0.17 | 0.00 | 0.14 | 0.21 | 0.08 | 0.15 | 0.25 | 22.7 |
| AM63 | UTCW | J25R | 53.91 | 0.91 | 0.76 | 0.00 | 0.00 | 0.13 | 0.02 | 0.04 | 0.04 | 74.8 |
| AM18 | UTCW | J25R | 56.27 | 0.96 | 0.92 | 0.00 | 0.00 | 0.04 | 0.00 | 0.02 | 0.00 | 25.1 |
| AM21 | UTCW | J25R | 57.51 | 0.88 | 0.90 | 0.00 | 0.00 | 0.05 | 0.02 | 0.02 | 0.01 | 49.2 |
| AM09 | UTCW | J25R | 57.91 | 0.95 | 0.91 | 0.00 | 0.00 | 0.04 | 0.02 | 0.02 | 0.01 | 69.9 |
| AM16 | UTCW | J25R | 61.00 | 0.88 | 0.52 | 0.02 | 0.08 | 0.10 | 0.07 | 0.08 | 0.14 | 26.2 |
| AMI01 | UTCW | J25R | 66.27 | 0.96 | 0.92 | 0.00 | 0.00 | 0.03 | 0.02 | 0.03 | 0.00 | 52.0 |
| AM70 | UTCW | J25R | 67.40 | 0.97 | 0.58 | 0.00 | 0.05 | 0.14 | 0.08 | 0.07 | 0.08 | 33.8 |
| AM19 | UTCW | J25R | 69.24 | 0.95 | 0.79 | 0.01 | 0.00 | 0.07 | 0.04 | 0.03 | 0.06 | 41.1 |
| AM67 | UTCW | J25R | 70.88 | 0.95 | 0.84 | 0.00 | 0.01 | 0.08 | 0.02 | 0.03 | 0.01 | 43.5 |
| AM13 | UTCW | J25R | 85.60 | 0.99 | 0.82 | 0.00 | 0.01 | 0.07 | 0.03 | 0.04 | 0.03 | 50.2 |
| AM15 | UTCW | J24RB | 88.01 | 0.91 | 0.09 | 0.00 | 0.00 | 0.48 | 0.17 | 0.23 | 0.04 | 114.3 |
| ***Umitaka Spur Northeast (UTNE)*** | | | | | | | | | | | | |
| AM23 | UTNE | J05R | 15.67 | 0.93 | 0.84 | 0.00 | 0.02 | 0.07 | 0.02 | 0.03 | 0.03 | 18.9 |
| AM22 | UTNE | J05R | 17.32 | 0.94 | 0.88 | 0.00 | 0.01 | 0.05 | 0.01 | 0.03 | 0.02 | 34.2 |
| AM37 | UTNE | J05R | 17.64 | 0.89 | 0.76 | 0.00 | 0.04 | 0.08 | 0.02 | 0.03 | 0.06 | 20.7 |
| AM66 | UTNE | J05R | 19.71 | 0.84 | 0.20 | 0.03 | 0.17 | 0.15 | 0.12 | 0.12 | 0.20 | 25.9 |
| AM60 | UTNE | J06RB | 50.00 | 0.91 | 0.66 | 0.00 | 0.06 | 0.09 | 0.04 | 0.04 | 0.11 | 73.5 |
|  |  |  |  |  |  |  |  |  |  |  |  |  |

**Table S2: Referenced Formulas and Calculations**

| **Calculation** | **Description** |
| --- | --- |
| **Ca:Mg ratio in microdolomite** | The Profex program^1^ was used to determine the cell parameters for the dolomite grains in each sample. A method employed by Turpin et al.^2^was then applied to determine the stoichiometric ratio of Mg:Ca in the dolomites in this study, assuming the general equation^2^:  Ca_(1+x)_Mg_(1-x)_(CO_3_)_2_. [1]  The Turpin method has an advantage of greater precision (±1.56% to ± 1.90%) over the more-traditional approach of calculating Mg:Ca from the position of the d_104_ peak (±4% to ±5%). This method first uses the Profex program to carry out Reitveld refinement (2) on the XRD patterns to determine the cell parameters of the dolomite, and then uses the relationship between a(=b) and c to calculate the relative amount of Ca and Mg such that^2^:  %CaCO_3_ = 279.92x -1295.00 for a(=b) [2]  where x is the a(=b) lattice parameter for in Å and %CaCO3 is the molar percentage in dolomite. Similarly, the dolomite composition was calculated using the c-lattice parameter^2^:  %CaCO3 = 48.99x -733.86 for c [3]  The presence of other divalent cations is assumed to be insignificant in this case, such that such that the sum of the molar fractions of Ca and Mg is assumed to be 100%. The two values derived from Eq.[2] and Eq.[3] were averaged for each sample and the stoichiometric ratio Mg:Ca was calculated according to Eq. [1] as:  Mg:Ca = (100%-%CaCO_3_)/(%CaCO3) [4]. |
| **Diameter of microdolomite grains** | Following image acquisition through both SEM and light microscopy, the average grain diameter of the microdolomites (Table S2) was determined by opening files in ImageJ^3^ and analyzing for the mean feret diameter using the MorphoLibJ plugin^3^. Where grains were overlapping, the underlying grain was deleted from the image prior to applying MorphoLibJ. Additionally, the microdolomite grains clumped as chains or “cauliflower” structures were analyzed as separate grains, such that the reported grain diameter represents an average ferret diameter for single and dumbbell-shaped aggregate grains. The number of grains analyzed for each sample ranged from 22-376 (avg.=158) and the reported diameters represent an average of the grain values for each sample. |
| **Equilibrium values for Stable isotopes** | The line representing the equilibrium value for δ^18^O relative to seawater in **Fig. 4c** was calculated by assuming an average geotherm value for Umitaka Spur of 0.105K/mbsf and a bottom-water temperature of 0.4^o^C^5^. The temperature relationship between dolomite and seawater is^6^:  Δ^18^O_DOL-PW_=1000lnα_DOL-PW_=2.73*10^6^/T^2^+0.26 [5]  Similarly, the line representing δ^13^C equilibrium for dolomite relative to measured CO_2_ shown in Fig. 4g assumes the same temperature gradient and the following relationship^7^:  Δ^12^C_DOL-CO2_=1000lnα_DOL-CO2_=1.637*10^6^/T^2^+7.29 [6] |

1. Lutterotti, L., Bortolotti, M., Ischia, G., Lonardelli, I. & Wenk, H.-R. Rietveld texture analysis from diffraction images. *Z. Kristallogr. Suppl*. **26**, 125-130 (2007).
2. Turpin, M., Nader, F.H. & Kohler, E. Empirical calibration for dolomite stoichiometry calculation: Application on Triassic Muschelkalk-Lettenkohle Carbonates (French Jura). *Pil & Gas Science and Technology: Rev. IFP Energies Nouvelles* **67**, 77-85 (2012).
3. Schneider, C.A., Rasband, W.S., Eliceiri, K.W. "NIH Image to ImageJ: 25 years of image analysis". *Nature Methods* **9**, 671-675, (2012).
4. Legland, D., Arganda-Carreras, I. & Andrey, P. MorphoLibJ: integrated library and plugins for mathematical morphology with ImageJ. *Bioinformatics***,** 3532-3534 (2016).
5. Machiyama, H. et al., 2009. Heat Flow Distribution around the Joetsu Gas Hydrate Field, Western Joetsu Basin, Eastern Margin of the Japan Sea. *Chigaku Zasshi (Journal of Geography)*, 118 (5), p. 986–1007, <https://doi.org/10.5026/jgeography.118.986> (2009).
6. Vasconcelos, C., McKenzie, J.A., Warthmann, R., & Bernasconi, S.M., Calibration of the δ^18^O paleothermometer for dolomite precipitated in microbial cultures and natural environments. *Geology* **33**, 317-320 (2005).
7. Horita, J. Oxygen and carbon isotope fractionation in the system dolomite-water-CO2 to elevated temperatures. *Geochimica et Cosmochimica Acta* **129**, 111-124 (2014).

**Table S3: Dolomite and Hydrate isotopic compositions**

|  | Sample Type | |  | Dol. |  | Dol |  | Hyd | Hyd |  |  |  |
| --- | --- | --- | --- | --- | --- | --- | --- | --- | --- | --- | --- | --- |
| ID | Location | Hole | mbsf | δ ^13^C |  | δ^18^O |  | H_2_S/CH_4_ | δ ^13^C _CO2_ | | Δ^13^C |  |
|  |  |  |  | ‰ |  | ‰ |  | (ml/L) | ‰ |  | _Dol-CO2_ |  |
| ***Joetsu Knoll (JK)*** | | | | | | | | | | | | |
| AM42 | JK | J04RB | 27.22 | 21.17 | ±0.08 | 1.20 | ±0.09 | 0.002 | n.d. |  | n.d. |  |
| ***Umitaka Spur Central West (UTCW)*** | | | | | | | | | | | | |
| AM28 | UTCW | J21R | 11.92 | 39.58 | ±0.03 | 2.91 | ±0.07 | 0.810 | 19.9 | ±0.3 | 19.7 |  |
| AM32 | UTCW | J24RB | 12.04 | 38.10 | ±0.07 | 3.59 | ±0.07 | 4.294 | 13.9 | ±0.3 | 24.2 |  |
| AM88 | UTCW | J21R | 12.22 | 39.33 | ±0.13 | 3.21 | ±0.08 | 0.663 | 22.0 | ±0.3 | 17.3 |  |
| AM31 | UTCW | J24RB | 14.12 | 39.95 | ±0.04 | 3.71 | ±0.07 | 2.538 | 17.8 | ±0.3 | 22.2 |  |
| AM71 | UTCW | J20R | 18.25 | 39.14 | ±0.51 | 4.78 | ±0.41 | n.d. | n.d. | n.d. | n.d. |  |
| AM10 | UTCW | J20R | 18.45 | 38.92 | ±0.09 | 3.91 | ±0.09 | 3.234 | 27.8 | ±0.3 | 11.1 |  |
| AM11 | UTCW | J20R | 19.93 | 39.66 | ±0.15 | 3.53 | ±0.53 | 3.114 | n.d. | n.d. | n.d. |  |
| AM68 | UTCW | J20R | 20.24 | 39.66 | ±0.42 | 3.34 | ±0.36 | 3.649 | 23.8 | ±0.3 | 15.9 |  |
| AM80 | UTCW | J22RC | 21.00 | 39.40 | ±0.53 | 3.61 | ±0.07 | 0.686 | 24.8 | ±0.5 | 14.6 |  |
| AM82 | UTCW | J22RC | 27.50 | 40.19 | ±0.42 | 4.04 | ±0.14 | 0.674 | 25.5 | ±0.3 | 14.7 |  |
| AM81 | UTCW | J22RC | 27.90 | 38.89 | ±0.17 | 3.10 | ±0.19 | 0.535 | 26.1 | ±0.5 | 12.8 |  |
| AM63 | UTCW | J25R | 53.91 | 37.00 | ±0.13 | 2.74 | ±0.18 | 1.766 | n.d. | n.d. | n.d. |  |
| AM18 | UTCW | J25R | 56.27 | 39.18 | ±0.07 | 4.04 | ±0.06 | 1.030 | n.d. | n.d. | n.d. |  |
| AM21 | UTCW | J25R | 57.51 | 36.16 | ±0.06 | 3.44 | ±0.00 | 1.232 | 21.3 | n.d. | 14.9 |  |
| AM09 | UTCW | J25R | 57.91 | 37.99 | ±0.04 | 4.02 | ±0.07 | 1.578 | 25.7 | ±0.3 | 12.3 |  |
| AM16 | UTCW | J25R | 61.00 | 36.75 | ±0.08 | 2.78 | ±0.09 | 0.232 | 25.1 | ±0.3 | 11.7 |  |
| AMI01 | UTCW | J25R | 66.27 | 41.23 | ±0.76 | 3.82 | ±0.15 | n.d. | n.d. | n.d. | n.d. |  |
| AM70 | UTCW | J25R | 67.40 | 39.32 | ±0.47 | 5.06 | ±0.34 | n.d. | n.d. | n.d. | n.d. |  |
| AM19 | UTCW | J25R | 69.24 | 40.46 | ±0.05 | 4.44 | ±0.03 | 1.898 | n.d. | n.d. | n.d. |  |
| AM67 | UTCW | J25R | 70.88 | 40.07 | ±0.23 | 3.16 | ±0.39 | 1.741 | 26.1 | ±0.3 | 14.0 |  |
| AM13 | UTCW | J25R | 85.60 | 41.63 | ±0.07 | 3.90 | ±0.38 | 0.010 | 29.3 | ±0.3 | 12.3 |  |
| AM15 | UTCW | J24RB | 88.01 | 36.74 | ±0.08 | 2.64 | ±0.13 | 0.005 | n.d. | n.d. | n.d. |  |
| ***Umitaka Spur Northeast (UTNE)*** | | | | | | | | | | | |  |
| AM23 | UTNE | J05R | 15.67 | 16.79 | ±0.05 | 4.11 | ±0.04 | 10.773 | n.d. | n.d. | n.d. |  |
| AM22 | UTNE | J05R | 17.32 | 18.04 | ±0.05 | 4.03 | ±0.05 | 10.523 | -0.7 | ±0.3 | 18.7 |  |
| AM37 | UTNE | J05R | 17.64 | 18.38 | ±0.07 | 3.90 | ±0.00 | 6.414 | 2.9 | 0.3 | 15.5 |  |
| AM66 | UTNE | J05R | 19.71 | 18.77 | ±0.40 | 1.94 | ±0.22 | 1.293 | n.d. | n.d. | n.d. |  |
| AM60 | UTNE | J06RB | 50.00 | 24.88 | ±1.12 | 1.38 | ±0.69 | 0.004 | n.d. | n.d. | n.d. |  |
|  |  |  |  |  |  |  |  |  |  |  |  |  |
|  |  |  |  |  |  |  |  |  |  |  |  |  |

**Table S4 Organic chemistry of dolomite-associated oils found in gas hydrate. Concentrations normalized to extractible organic substances (EOS) and to asphaltene (asph.)**

| ID | Location | Hole | mbsf | *Asphaltene (ug/mg) | C_29_ αβ 25-Norhopane +(ug/mg-EOS) | C_29_ αβ 25-Northopane +(ug/mg-asph.) | C_30_ αβ Hopane +(ug/mg-EOS) | C_30_ αβ Hopane +(ug/mg-asph.) | Sum C27,28 & 29 Diasterane +(ug/mg-EOS) | Sum C27,28 & 29Diasterane +(ug/mg asph) |
| --- | --- | --- | --- | --- | --- | --- | --- | --- | --- | --- |
| **Joetsu Knoll (JK)** | | | | | | | | | | |
| AM42 | JK | J04RB | 27.22 | n.d. | n.d. | n.d. | n.d. | n.d. | n.d. | n.d. |
| **Umitaka Spur Central West (UTCW)** | | | | | | | | | | |
| AM28 | UTCW | J21R | 11.92 | n.d. | n.d. | n.d. | n.d. | n.d. | n.d. | n.d. |
| AM32 | UTCW | J24RB | 12.04 | n.d. | n.d. | n.d. | n.d. | n.d. | n.d. | n.d. |
| AM88 | UTCW | J21R | 12.22 | n.d. | n.d. | n.d. | n.d. | n.d. | n.d. | n.d. |
| AM31 | UTCW | J24RB | 14.12 | n.d. | n.d. | n.d. | n.d. | n.d. | n.d. | n.d. |
| AM71 | UTCW | J20R | 18.25 | 78.571 | 0.008 | 0.105 | 0.015 | 0.195 | 0.023 | 0.294 |
| AM10 | UTCW | J20R | 18.45 | n.d. | n.d. | n.d. | n.d. | n.d. | n.d. | n.d. |
| AM11 | UTCW | J20R | 19.93 | n.d. | n.d. | n.d. | n.d. | n.d. | n.d. | n.d. |
| AM68 | UTCW | J20R | 20.24 | n.d. | n.d. | n.d. | n.d. | n.d. | n.d. | n.d. |
| AM80 | UTCW | J22RC | 21.00 | 53.571 | 0.008 | 0.152 | 0.035 | 0.627 | 0.036 | 0.671 |
| AM82 | UTCW | J22RC | 27.50 | 47.778 | n.d. | n.d. | n.d. | n.d. | n.d. | n.d. |
| AM81 | UTCW | J22RC | 27.90 | 20.000 | 0.007 | 0.373 | 0.014 | 0.756 | 0.021 | 1.027 |
| AM63 | UTCW | J25R | 53.91 | 148.667 | 0.009 | 0.063 | 0.029 | 0.133 | 0.036 | 0.244 |
| AM18 | UTCW | J25R | 56.27 | n.d. | n.d. | n.d. | n.d. | n.d. | n.d. | n.d. |
| AM21 | UTCW | J25R | 57.51 | n.d. | n.d. | n.d. | n.d. | n.d. | n.d. | n.d. |
| AM09 | UTCW | J25R | 57.91 | 97.000 | 0.011 | 0.116 | 0.031 | 0.319 | 0.025 | 0.263 |
| AM16 | UTCW | J25R | 61.00 | n.d. | n.d. | n.d. | n.d. | n.d. | n.d. | n.d. |
| AMI01 | UTCW | J25R | 66.27 | n.d. | n.d. | n.d. | n.d. | n.d. | n.d. | n.d. |
| AM70 | UTCW | J25R | 67.40 | 262.500 | 0.015 | 0.058 | 0.031 | 0.109 | 0.028 | 0.108 |
| AM19 | UTCW | J25R | 69.24 | 58.333 | 0.012 | 0.198 | 0.013 | 0.205 | 0.025 | 0.424 |
| AM67 | UTCW | J25R | 70.88 | 480.000 | 0.018 | 0.037 | 0.024 | 0.067 | 0.043 | 0.089 |
| AM13 | UTCW | J25R | 85.60 | n.d. | n.d. | n.d. | n.d. | n.d. | n.d. | n.d. |
| AM15 | UTCW | J24RB | 88.01 | 285.714 | 0.016 | 0.057 | 0.022 | 0.103 | 0.044 | 0.153 |
| **Umitaka Spur Northeast (UTNE)** | | | | | | | | | | |
| AM23 | UTNE | J05R | 15.67 | n.d. | n.d. | n.d. | n.d. | n.d. | n.d. | n.d. |
| AM22 | UTNE | J05R | 17.32 | n.d. | n.d. | n.d. | n.d. | n.d. | n.d. | n.d. |
| AM37 | UTNE | J05R | 17.64 | n.d. | n.d. | n.d. | n.d. | n.d. | n.d. | n.d. |
| AM66 | UTNE | J05R | 19.71 | n.d. | n.d. | n.d. | n.d. | n.d. | n.d. | n.d. |
| AM60 | UTNE | J06RB | 50.00 | n.d. | n.d. | n.d. | n.d. | n.d. | n.d. | n.d. |
|  |  |  |  |  |  |  |  |  |  |  |

*Asphaltene was determined using surface enhanced Raman spectroscopy. + Quantities are reported relative to an internal standard of 5 β-cholane.

**Table S5: Phylogenetic distribution determined by 16s mRNA analysis for the internal contents of four microdolomite samples.**

|  | J24RB  (12.04 mbsf) | J20R  (18.25 mbsf) | J25R  (53.91 mbsf) | J25R  (57.91mbsf) |
| --- | --- | --- | --- | --- |
| α-proteobacteria | 12.2% | 17.9% | 0.0% | 0.0% |
| *Rhizobiales* | 1.5% | 0.7% | 43.4% | 0.0% |
| *Sphingomonadales^*^* | 16.5% | 28.7% | 45.8% | 55.6% |
| β-proteobacteria | 0.0% | 0.2% | 0.0% | 0.0% |
| *Burkholderiales^*^* | 0.0% | 0.0% | 4.2% | 0.0% |
| *Hydrogenophilales^*^* | 0.0% | 0.2% | 0.0% | 0.0% |
| γ-proteobacteria | 2.4% | 1.2% | 0.0% | 0.0% |
| δ-proteobacteria | 0.0% | 0.5% | 0.0% | 0.0% |
| Actinobacteria | 0.0% | 2.1% | 6.6% | 8.4% |
| Bacteroidetes | 0.7% | 0.9% | 0.0% | 0.0% |
| *Flavobacteriia^#^* | 48.3% | 25.3% | 0.0% | 0.0% |
| Chloroflexi | 0.0% | 0.0% | 0.0% | 0.0% |
| Cyanobacteria | 17.0% | 9.1% | 0.0% | 0.0% |
| Firmicutes | 0.0% | 11.8% | 0.0% | 0.0% |
| *Bacilli* | 0.2% | 0.1% | 0.0% | 0.0% |
| *Thermodesulfovibrionia ^#^* (Nitrospirae) | 1.1% | 0.8% | 0.0% | 9.8% |
| Other Bacteria | 0.2% | 0.1% | 0.0% | 26.2% |
| Euryarchaeota (Archaea) | 0.0% | 0.5% | 0.0% | 0.0% |

*Order, *^#^*Class.
